# The Biological and Clinical Role of the Long Non-Coding RNA LOC642852 in Ovarian Carcinoma

**DOI:** 10.3390/ijms21155237

**Published:** 2020-07-23

**Authors:** Natalie Filippov-Levy, Reuven Reich, Ben Davidson

**Affiliations:** 1 Institute of Drug Research, School of Pharmacy, Faculty of Medicine, The Hebrew University of Jerusalem, Jerusalem 91120, Israel; natalie_sunday@yahoo.com; 2 Institute of Clinical Medicine, Faculty of Medicine, University of Oslo, N-0316 Oslo, Norway; bend@medisin.uio.no; 3 Department of Pathology, Oslo University Hospital, Norwegian Radium Hospital, N-0310 Oslo, Norway

**Keywords:** LOC642852, long non-coding RNA, CRISPR/Cas9, ovarian carcinoma, tumorigenesis

## Abstract

The objective of the present study was to analyze the biological and clinical role of the long non-coding RNA LOC642852 in ovarian carcinoma (OC). LOC642852 expression was analyzed in seven OC cell lines (OVCAR-3, OVCAR-8, OVCA 433, OVCA 429, OC 238, DOV13, ES-2) and 139 high-grade serous carcinoma (HGSC) specimens (85 effusions, 54 surgical specimens). Following LOC642852 knockout (KO) using the CRISPR/Cas9 system, OVCAR-8 HGSC cells were analyzed for spheroid formation, migration, invasion, proliferation, matrix metalloproteinase (MMP) activity, and expression of cell signaling proteins. OVCAR-8 cells with LOC642852 KO were significantly less motile and less invasive compared to controls, with no differences in spheroid formation, proliferation, or matrix metalloproteinase (MMP) activity. Total Akt and Erk levels were comparable in controls and KO cells, but their phosphorylation was significantly increased in the latter. In clinical specimens, LOC642852 was overexpressed in ovarian tumors and omental/peritoneal metastases compared to effusion specimens (*p* = 0.013). A non-significant trend for shorter overall (*p* = 0.109) and progression-free (*p* = 0.056) survival was observed in patients with HGSC effusions with high LOC642852 levels. Bioinformatics analysis showed potential roles for LOC642852 as part of the TLE3/miR-221-3p ceRNA network and in relation to the FGFR3 protein. In conclusion, LOC642852 inactivation via CRISPR/Cas9 affects cell signaling, motility, and invasion in HGSC cells. LOC642852 is differentially expressed in HGSC cells at different anatomical sites. Its potential role in regulating the TLE3/miR-221-3p ceRNA network and FGFR3 merits further research.

## 1. Introduction

Only a small part of the genome encodes proteins, while the rest is transcribed to non-coding RNA. Long non-coding RNAs (lncRNAs) are non-coding RNAs containing more than 200 nucleotides. LncRNA genes have protein-coding characteristic chromatin marks (e.g., H3K4me3 at promoters) and are transcribed and processed as mRNA, undergoing splicing, capping, and polyadenylation [1]. In recent years, several functions of lncRNAs have been discovered, including chromatin regulation, mRNA decoy, regulation of protein activity, and scaffolding. LncRNA are additionally precursors for small RNA and miRNA, and function as sponges or part of competing endogenous RNAs (ceRNAs), a complex interaction network between mRNA, lncRNA, and microRNA [2]. In cancer, lncRNA have been found to undergo mutation and have aberrant expression [3].

Ovarian cancer is the most aggressive gynecologic cancer, consisting predominantly (>90%) of ovarian carcinoma (OC). The majority (70%) of OCs are high-grade serous carcinomas (HGSC).

In 2019, ovarian cancer was predicted to be diagnosed in 22,530 women and result in 13,980 fatalities in the US [4]. Globally, 295,414 women were diagnosed with the disease, and 184,799 died of it in 2018, making this cancer the eighth most common and eighth most lethal malignancy in women [5]. Late diagnosis, when the disease is at an advanced stage, and chemotherapy resistance are two major factors contributing to the fact that the five-year survival in OC is below 50%, with the majority of patients succumbing to the disease [6]. In OC, lncRNAs have been shown to have a role in many critical pathways, including invasion, proliferation, cell cycle, apoptosis, and drug resistance [7,8,9].

The present study analyzed the biological and clinical role of the lncRNA LOC642852 (also known as LINC00205 or NR_026943.1) in OC cell lines and clinical specimens.

## 2. Results

### 2.1. LOC642852 in OC Cell Lines

**Figure 1:**
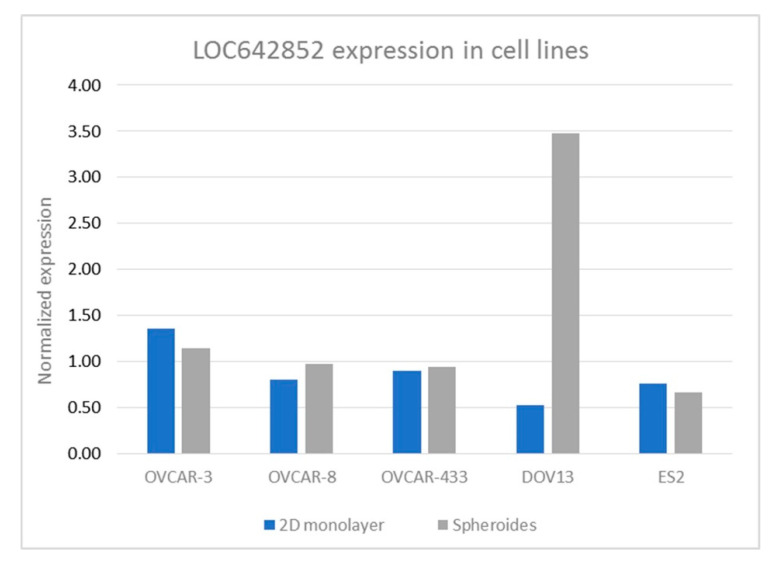
LOC642852 expression in different cell lines cultured in 2D monolayers or spheroids.

We analyzed the expression of LOC642852 in seven cell lines: OVCAR-3, OVCAR-8, OVCA 429, OVCA 433, OC-238, DOV13, and ES2 under two culture conditions—monolayer cells and spheroid form (Figure 1). No expression of LOC642852 was found in OC-238 and OVCA 429 cells irrespective of the culture condition. The CRISPR/Ca9 KO was performed on the OVCAR-8, ES-2 and DOV13 cell lines. KO was successful in OVCAR-8 and ES-2 and proved lethal for DOV13 cells. OVCAR-8 was chosen for the in vitro experiments since it was an HGSC line, in agreement with the histology of the clinical specimens studied, with three KO lines formed, labeled G9(3), B9(1), and C10(4) (Figure S1).

### 2.2. LOC642852 KO Affects Invasion and Migration, but Not MMP Activity, Proliferation or Spheroid Formation

**Figure 2:**
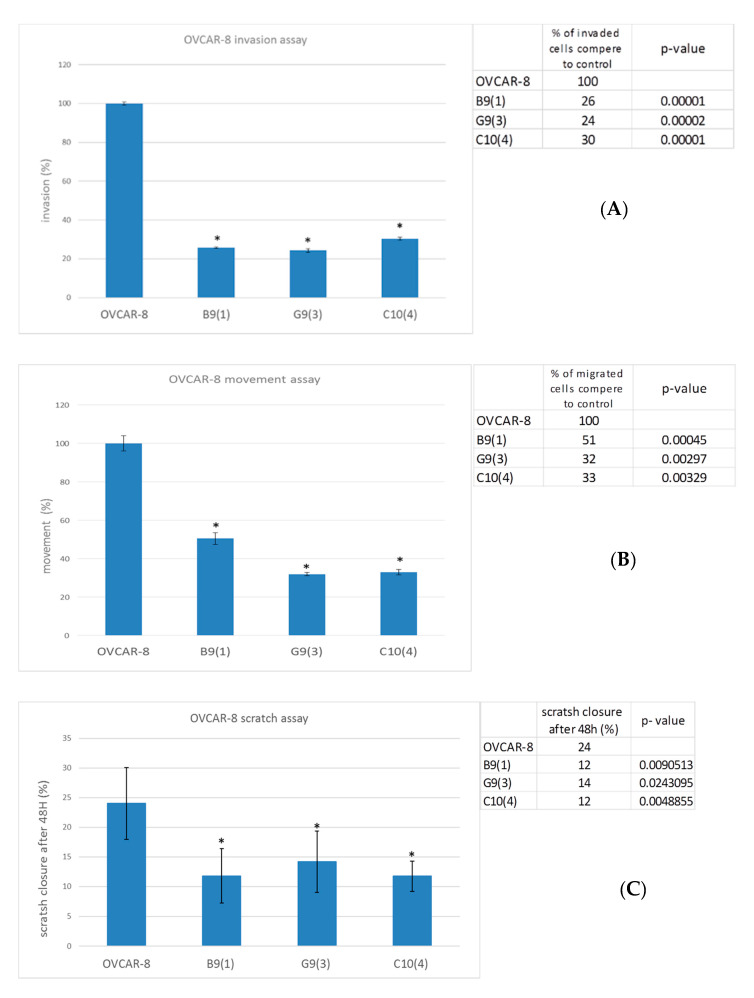

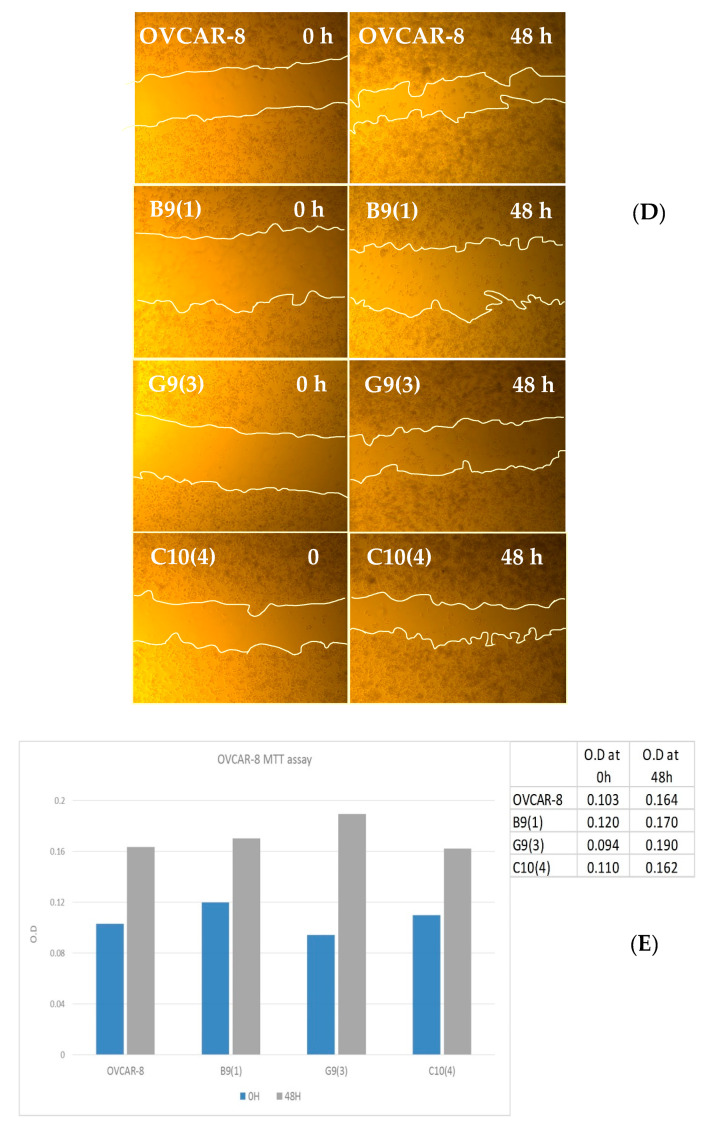
(**A**) Graph and table showing the percentage and number of OVCAR-8 KO cells that migrated through the matrigel compared to the control cells, with a *p*-value for each line (* = significant difference). (**B**) Graph and table showing the percentage and number of OVCAR-8 KO cells that migrated throughout collagen IV compared to control cells, with a *p*-value for each line (* = significant difference). (**C**,**D**) Wound healing assay. Graph, table, and images showing the percentage of the scratch closed by KO cells and OVCAR-8 control cells, with a *p*-value for each line (* = significant difference). (**E**) MTT assay. A graph showing the optical density O.D. of OVCAR-8 KO and control cells at 0 and 48 h (*p* > 0.05).

To assess the role of LOC542852 in cell migration, we performed Matrigel invasion assay for 5 and 24 h. The ability of the G9(3), B9(1), and C10(4) KO to invade was severely impaired, with reduction to 24, 26 and 30% of the control value, respectively (Figure 2A). Similarly, cell motility was significantly reduced in all KO lines compared to controls, with values at 51, 32 and 33% for the G9(1), G9(3), and C10(4) lines, respectively (Figure 2B). A similar result was also obtained in a wound healing assay, where B9(1) and C10(4) cells closed 12% and G9(3) 14% of the gap, whereas control cells closed 24% (Figure 2C,D).

Zymography did not show any difference in MMP2 and MMP9 secretion between LOC642852 KO cells and controls.

To analyze whether LOC642852 KO affects proliferation, we performed the MTT assay. No significant difference was found between the number of control and KO cells at 48 h (Figure 2E).

Another parameter assessed was the ability of the cells to form spheroids. No difference in spheroid number, shape, or size was observed between KO and the control cells after 24 or 48 h.

### 2.3. LOC642852 and Cell Signaling

**Figure 3:**
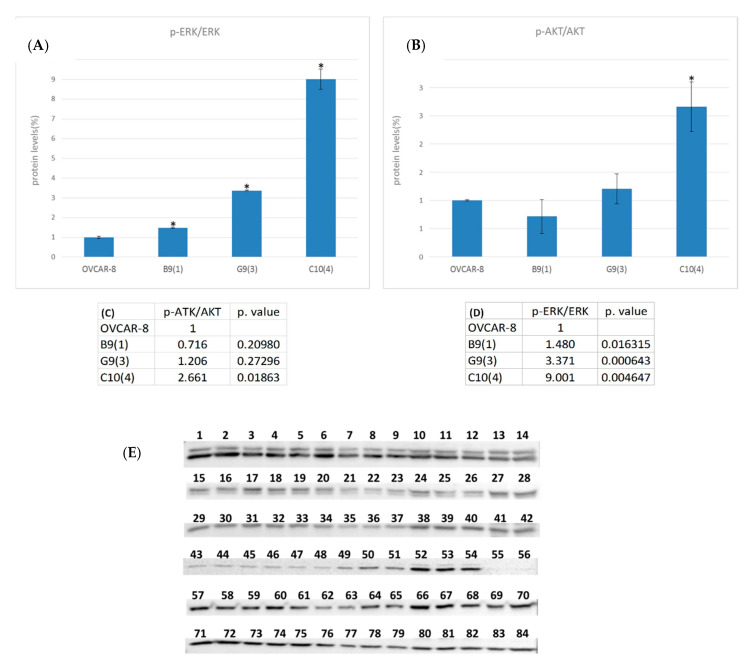
(**A**,**B**) *p*-EKR/ERK and *p*-AKT/AKT protein levels (after normalization to Glyceraldehyde 3-phosphate dehydrogenase GAPDH) with Western-blot (WB) images (**E**) below the graph (*-significant *p*-value). (**C**,**D**)—detailed table of *p*-EKR/ERK and *p*-AKT/AKT results and the *p*-value. (**E**) detailed WB of the various samples.

To explore the effect of LOC642852 KO on cell signaling, we examined the change in ERK1/2 and AKT protein expression and phosphorylation status. No change in ERK1/2 expression level was observed, but significant up-regulation in the phosphorylation was observed in all KO lines compared to controls. For AKT, significant upregulation in phosphorylation was found in C10(4) KO (Figure 3 and Table 1).

### 2.4. LOC642852 is Overexpressed in Solid Specimens Compared to HGSC Effusions

**Figure 4:**
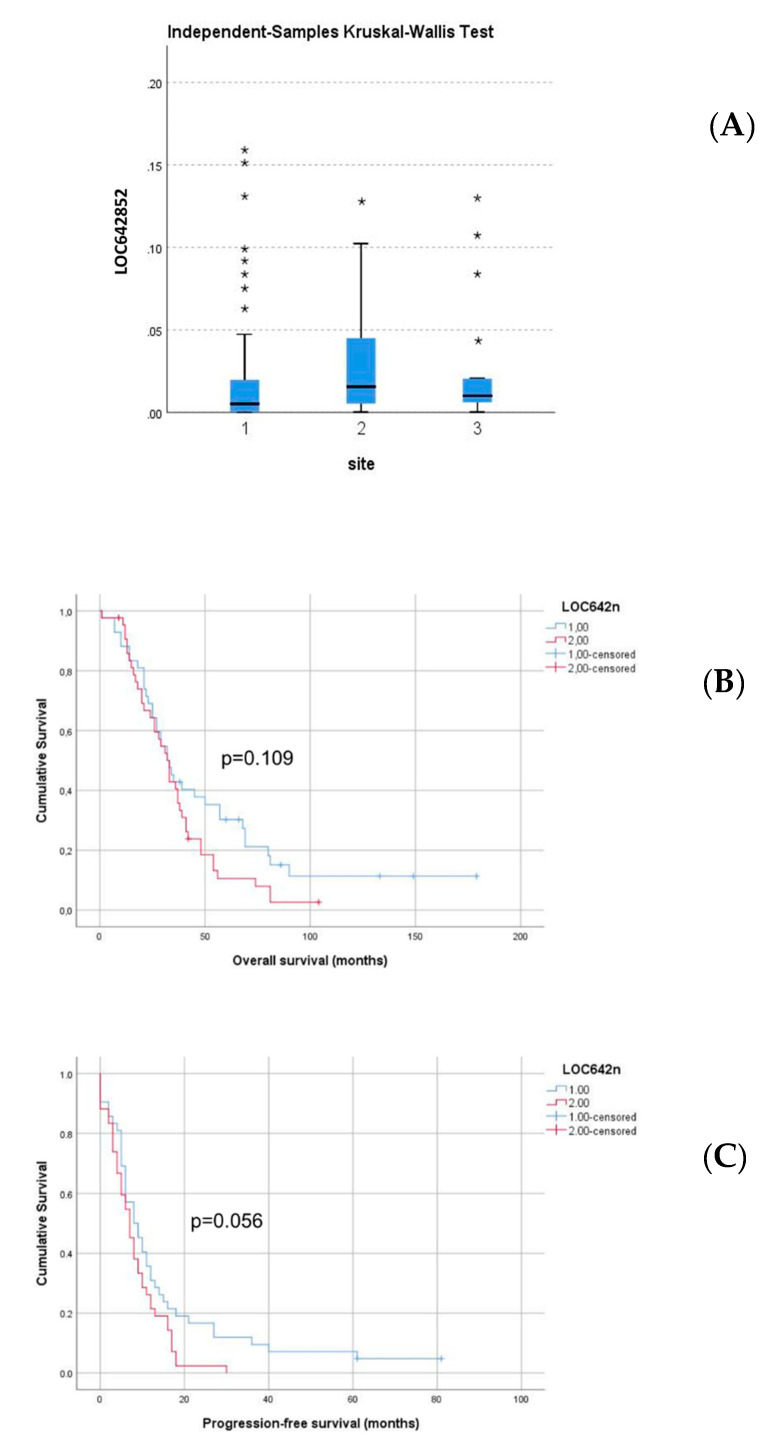
Anatomic site-related expression and Survival analysis (**A**) LOC642852 is overexpressed in the ovarian tumors (marked ‘2′) and solid metastases (marked ‘3′) compared to effusion specimens (marked ‘1′; *p* = 0.013) * -value of individual samples (**B**) Kaplan-Meier survival curve showing the association between LOC642852 expression level and overall survival (OS) for 85 patients with HGSC effusions. Patients with effusions with higher-than-median LOC642852 expression level (*n* = 43; red line) had mean OS of 35 months compared to 53 months for patients with effusions having lower-than-median expression level (*n* = 43, blue line; *p* = 0.109). (**C**) Kaplan–Meier survival curve showing the association between LOC642852 expression level and progression-free survival (PFS) for 84 patients with HGSC effusions (one patient with missing PFS data). Patients with effusions with higher-than-median LOC642852 expression level (*n* = 42; red line) had mean PFS of 15 months compared to 8 months for patients with effusions having lower-than-median expression level (*n* = 42, blue line; *p* = 0.056).

A comparative analysis of surgical specimens and effusions from HGSC patients showed overexpression of this lncRNA in ovarian tumors and omental/peritoneal metastases compared to effusion specimens (mean rank = 85.40, 78.44 and 62.18, respectively; *p* = 0.013; Figure 4A).

In the effusion cohort, LOC642852 levels were unrelated to effusion site, prior chemotherapy, age, Federation of Gynecology and Obstetrics (FIGO) stage, residual (RD) volume, or chemotherapy response.

The follow-up period for patients with HGSC effusions ranged from 1 to 179 months (mean = 40 months, median = 32 months). The Progression-free survival (PFS) ranged from 0 to 81 months (mean = 11 months, median = 8 months). At the last follow-up, 75 patients were dead from the disease, seven were alive with the disease, and one had no evidence of the disease. One patient died of complications and one was lost to follow-up. Patients with higher-than-median LOC642852 levels had shorter overall survival (OS) and PFS in univariate survival analysis, though not significantly so (OS: 35 vs. 53 months; *p* = 0.109; Figure 4B; PFS: 8 vs. 15 months; *p* = 0.056; Figure 4C).

We additionally examined the association between LOC642852 levels and survival using the Kaplan–Meier Plotter web tool (https://kmplot.com/analysis/index.php?p=service), dataset 22,8908_s_at, with available data on OS and PFS for 655 and 614 patients, respectively. In this dataset, patients with high (above median) LOC642852 expression had a median OS of 36.83 months compared to 46.82 months for patients with low (below median) LOC642852 expression (*p* = 0.0056). PFS for patients with high LOC642852 expression was 12.83 months compared to 19 months for patients with low (below median) LOC642852 expression (*p* = 0.00026) (Figure S2).

### 2.5. Identification of the Potential Biological Role of LOC642852 in OC

As LOC642825, to the best of our knowledge, is a novel lncRNA, we explored different pathways through which it may act.

#### 2.5.1. LOC642852/TLE3 ceRNA Network

**Figure 5:**
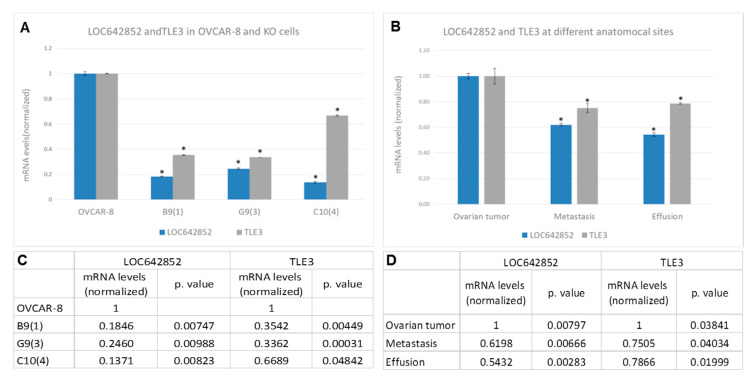
LOC642852 partners. (**A**) Graph showing mRNA levels of LOC642852 and family member 3, transcriptional corepressor (TLE3) in OVCAR-8 control cells and KO cells. (**B**) Graph showing mRNA levels of LOC642852 and TLE3 at different anatomical sites in clinical ovarian cancer (OC) specimens. Each site is a pool of 24 patient samples (* significant *p*-value). (**C**,**D**) detailed table of mRNA expression levels and *p*-values for graphs A and B, respectively.

We searched for potential miRs binding LOC642825 using the RNA22 tool (https://cm.jefferson.edu/rna22/), miRCODE (www.mircode.org/), and microRNA.org—Targets and Expression (http://www.microrna.org/microrna/getGeneForm.do). A total of 52 miRs were significantly predicted to bind to LOC682852 between bases 1115–1150, of which 2—miR-221-3p and miR-222-3p—were found in our previous study to be overexpressed in OC effusions compared to the tumor in the ovary [10]. Additionally, for the 52 miRs, a search for mRNA targets using the miRBD website (http://mirdb.org/) was done, in which a total of 1626 genes were found. LOC642852 expression was correlated side-by-side with all mRNA transcripts found in the same microarray in which LOC642852 was identified. The top 500 were compared to the 1626 genes, and 35 shared genes were identified. Of them, the family member 3, transcriptional corepressor (TLE) family member 3, transcriptional corepressor (TLE3), was found to have a binding site to miR-221-3p, as well as the highest correlation to LOC642852, a correlation score of -0.987. We, therefore, assumed a ceRNA relation between TLE3 and LOC642852. TLE3 expression in KO cells reinforced our hypothesis, as we found significant down-regulation of TLE3 in all KO lines (Figure 5A,C). Furthermore, we examined TLE3 and LOC642852 expression in pools of OC samples from ovarian tumors, omental/peritoneal metastases, and effusions. For both, the highest expression was found in ovarian tumors compared to solid metastases and effusion specimens (Figure 5A,C).

#### 2.5.2. LOC642852 and FGFR3

The top 500 correlated genes were subjected to pathways analysis, and enrichment of FGFR3 pathways was found. As FGFR3 and LOC642852 do not have shared miRs, and as FGFR3 mRNA levels did not differ between control and KO cells (Figure S3), we hypothesized that they might interact at the protein level. To examine this hypothesis, we used the “Global score” website (http://service.tartaglialab.com/update_submission/199876/0ebb81064c), a platform that enabled us to evaluate the probability of LOC642852 and FGFR3 to interact. A possible interaction between the bases 3034–3321, 3576–3863, and 3606–3893 of LOC642852 and 476–527aa, 550–601aa, and 650–70aa of FGFR3 was found. On the FGFR3 protein, these sites were the ATP-binding site and the activation loop.

## 3. Discussion

Recent research has documented the biological role of lncRNAs in OC [7,8,9], and several studies have demonstrated an association between lncRNA expression and patient survival in this cancer [11,12]. There are currently between 32 and 58 lncRNAs that have been reported to have a role in OC pathogenesis [7,8,9].

**Figure 6:**
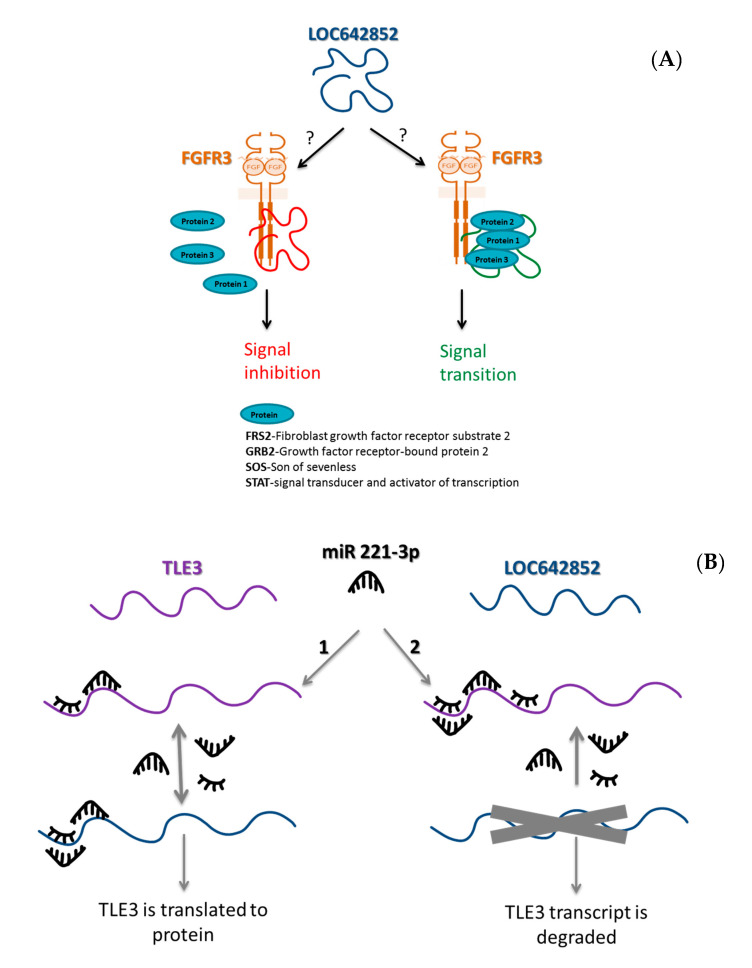
LOC642852 interactions (**A**) The lncRNA LOC642852 may serve as an inhibitor of signaling for FGFR3 by preventing crucial proteins, such as signal transducer and activator of transcription (STAT) and Fibroblast Growth Factor Receptor Substrate 2 (FRS2) to access the receptor, or alternatively, as a scaffold for the assembly of different proteins such as FRS2, Growth Factor Receptor Bound Protein 2 (GRB2), Son of Sevenless (SOS), and STAT. (**B**) LOC642852, TLE3, and miR-221-3p ceRNA network. At option (1), both transcripts are expressed, miR-221-3p is divided between them, and TLE3 is translated to a protein. At option (2), when LOC642852 is not expressed, all miRs are attached to TLE3, causing its degradation.

The present study is, to the best of our knowledge, the first one exploring the expression, biological role, and clinical relevance of LOC642852 in OC. LOC642852 KO decreased invasion and motility, but had no effect on MMP secretion, proliferation, and spheroid formation. In addition, an increase in ERK1/2 and AKT phosphorylation was observed in LOC642852 KO cells. The latter was most pronounced in the C10(4) KO, possibly due to the fact that its LOC642852 expression level was the lowest among all KOs. Using bioinformatics tools, two potential roles for LOC642852 were identified, i.e., interactions with FGFR and TLE3. FGFR3 is a member of the FGFR receptor tyrosine kinase family, which belongs to the immunoglobulin (Ig) superfamily. FGFR signaling is via the PI3K/AKT, STAT, and RAS/MAPK pathways [13]. We hypothesize that LOC642852 may serve as a platform for protein interaction near FGFR3, thus enabling downstream signaling. Alternatively, this interaction may be of an inhibitory nature, in which case, LOC642852 would prevent the assembly of FGFR3-related proteins (Figure 6A).

The second potential interaction may be between LOC642852, TLE3, and miR-221-3p (Figure 6B). TLE3 is a transcriptional co-repressor protein involved in cell differentiation and tumorigenesis [14] and was reported to be a potential marker for responsiveness to taxane treatment in OC [15]. Our bioinformatics analysis identified miR-221-3p binding sites on both LOC642852 and TLE. Moreover, differences in the levels of these between HGSC specimens at different anatomical sites, as well as in KO compared to control OVCAR-8 cells, corresponded. These results lead us to hypothesize that LOC642852 and TLE, along with miR-221-3p, are a ceRNA network. In recently published studies of hepatocellular carcinoma (HCC), LINC00205 was found to interact with miR-122-5p to promote proliferation, migration, and invasion, and with miR-184 to modulate epoxide hydrolase 1 (EPHX1) expression [16,17]. This suggests a biological role for this lncRNA in cancer.

Data regarding the prognostic role of LINC00205/ LOC642852 are inconclusive at present and may be organ-dependent. LINC00205 was identified as a marker of short relapse-free survival in HCC [18]. Similarly, higher expression of this lncRNA, designated LOC642852, as part of an 8-lncRNA panel, was significantly associated with shorter OS in colorectal cancer [19]. In contrast, LINC00205 overexpression in pancreatic cancer was significantly associated with better survival [20]. In the present study, LOC642852 levels in HGSC effusions were not significantly related to chemoresponse or to survival, although a trend towards shorter OS and PFS was observed. An assessment of the clinical relevance of this lncRNA in surgical specimens from patients with HGSC, or in other OC histotypes, awaits future studies.

In summary, the present study identifies two potential functions at the mRNA and protein levels for LOC642852 in HGSC, as well as anatomical site-dependent expression of this lncRNA in this tumor. Despite the lack of prognostic relevance in this cohort, further research into the biological role of this molecule may yield knowledge regarding cellular pathways that may be targeted in this malignancy.

## 4. Materials and Methods

### 4.1. Cell Culture

OC cell lines included in this study consisted of OVCAR-3 (HGSC), OVCAR-8 (HGSC), OVCA 433 (serous adenocarcinoma), OVCA 429 (cystadenocarcinoma), OC 238 (serous cystadenocarcinoma), DOV13 (adenocarcinoma), and ES-2 (clear cell carcinoma). All cell lines were cultured in 37 °C, 5% CO_2_ in the following media: OVCAR-3, OC-238, ES-2—Dulbecco’s Modified Eagle’s medium-high glucose (DMEM; Sigma-Aldrich, St. Louis, MO, USA) 10% fetal calf serum (FCS; Sigma-Aldrich). OVCA 433, OVCA 429, DOV13—MEM-EAGLA Earle’s Salts Base (MEM; Biological Industries, Kibbutz Beit-Haemek, Israel) 10% FCS. OVCAR-8: RPMI Medium 1640 (GIBCO, Thermo Fisher Scientific, Waltham, MA, USA) 5% FCS. All media were supplemented with 1% MEM-Eagle non-essential amino acids solution, 1% l-Glutamine Solution, 1% MEM Vitamins Solution, 1% Sodium Pyruvate Solution, 1% Penicillin-Streptomycin-Amphotericin B Solution (Biological Industries, Kibbutz Beit Haemek, Israel).

### 4.2. Patients and Specimens

HGSC effusions (*n* = 85; 70 peritoneal, 15 pleural) from 85 patients were submitted to the Department of Pathology at the Norwegian Radium Hospital during the period of 1998 to 2015. The effusions were centrifuged immediately after tapping, and cell pellets were used for the preparation of cellblocks using the thrombin clot protocol. Cell pellets were additionally frozen at −70 °C in equal amounts of RPMI 1640 medium (GIBCO) containing 50% fetal calf serum (PAA Laboratories GmbH, Pasching, Austria) and 20% dimethylsulfoxide (Merck KGaA, Darmstadt, Germany). Tumor cell content in all effusions studied by Western blotting was >50%, based on the assessment of cytology smears and H&E sections from the above cellblocks, as well as immunohistochemistry, based on current guidelines [21]. The clinicopathologic data are detailed in Table 2.

A series of 54 HGSC surgical specimens was analyzed for comparative purposes. These consisted of 30 tumors localized to the ovary and 24 omental/peritoneal metastases. As the fallopian tube was not assessed based on current guidelines in this series, ovarian tumors are referred to as such, with no designation as primary vs. metastatic focus. An HGSC diagnosis was made based on a combination of morphology and p53 staining patterns, according to current guidelines [22]. Frozen sections from all solid tumors were reviewed by an experienced gynecopathologist (BD), and only specimens with tumor cell population >50% and minimal or no necrosis were included.

Informed consent was obtained according to national and institutional guidelines. The study approval was given by the Regional Committee for Medical Research Ethics in Norway (Ethics approval S-04300).

### 4.3. CRISPR/Cas9 KO

#### 4.3.1. Guides

As we did not know which part of the lncRNA was crucial for its function, we decided to delete the first exon. To achieve this goal, we designed two guides that cut out 145 of the 193 bases in the first exon. The guides were designed with the help of the MIT (https://zlab.bio/guide-design-resources) and UCSC (http://genome.ucsc.edu/index.html) websites. Each guide was cloned into a PX330 plasmid (pX330-U6-Chimeric_BB-CBh-hSpCas9; a kind gift from Yosef Buganim’s lab, Hebrew University).

#### 4.3.2. Transfection

PX330 plasmid transfection into OVCAR-8 cells was made using Avalanche-Everyday (EZ Biosystems, College Park, MD, USA). A total of 1.25 μg of each guide plasmid and 0.5 μg of the puromycin-resistant plasmid (Fuw-original-puro-2A-EGFP was a kind gift from Yosef Buganim’s lab) were co-transfected for 24 h, followed by puromycin selection for another 48 h. The transfected cells were then split into single cells in a 96-well plate.

### 4.4. Spheroid Formation

OVCAR-8 cells (*n* = 400,000) were seeded on a six-well plate dish and agitated for 24 or 48 h, followed by imaging and medium collection for RNA isolation.

### 4.5. Scratch Assay

OVCAR-8 cells (*n* = 100–200,000) were seeded on a 12-well plate dish and cultured to confluence. A scratch was made with a 1000 μL pipette tip, and images were taken at 0, 6, 24, and 48 h. The results were analyzed using the ImageJ program (NIH, Bethesda, MD, USA).

### 4.6. Cell Proliferation Assay

OVCAR-8 cells (*n* = 100,000) were seeded on a 12-well plate. At 0 and 48 h, the cells were treated with 2 mg/mL of 3-[4,5-dimethylthiazole-2-yl]-2,5-diphenyltetrazolium bromide (MTT; Calbiochem, San Diego, CA, USA) for 20 min at 37 °C in a 5% CO_2_ incubator. Cells were subsequently lysed using 150 μL Dimethyl Sulfoxide (DMSO; Sigma-Aldrich) and absorption at 570 nm was measured by a Cytation 3 instrument (BioTek Instruments, Inc., Winooski, VT, USA).

### 4.7. Matrigel Invasion Assay and Motility Assay

Nuclepore polycarbonate filters (13 mm, 8 μm pore size, polyvinylpyrrolidone [PVP]-free; Whatman International Ltd., Maidstone, UK) were coated with basement membrane components extract (Matrigel, 25 μg per filter) for the chemo-invasion assay or with 5 μg collagen IV for the motility assay, and placed in Boyden chambers. OVCAR-8 cells (*n* = 200,000) were resuspended in a serum-free medium and placed in the upper compartment of the Boyden chambers. A chemoattractant, fibroblast-conditioned medium (obtained from NIH-3T3 cells) was used. After 5 or 24 h of incubation at 37 °C in a 5% CO_2_ incubator, the filters’ lower surface was stained in DiffQuik (Medion Diagnostics International Inc., Miami, FL, USA) and five random fields were counted.

### 4.8. Matrix Metalloproteinase (MMP) Activity Assay (Zymography)

The assay was performed a medium collected from Boyden chambers at the end of the Matrigel invasion experiment. Media from OVCAR-8 controls and KO cells were loaded onto 10% sodium-dodecyl-sulfate (SDS)-Polyacrylamide gel electrophoresis (PAGE) gels with 1 mg/mL gelatin (Sigma-Aldrich), as previously described [23].

### 4.9. Western Blot

The total protein isolation was performed using RIPA (1% NP-40, 20 mM Tris-HCl (pH 7.5), 137 mM NaCl, 0.5 mM EDTA, 10% glycerol), 1% protease inhibitor cocktail (Millipore, Burlington, MA, USA), 1 mM sodium Orthovanadate, and 0.1% sodium dodecyl sulfate (SDS). 25 μg of protein was loaded onto 10% SDS-PAGE gel. The proteins were then transferred to an Immobilon PVDF membrane (Millipore). Subsequently, the membrane was blocked by 5% Difco skims milk (BD Biosciences, San Jose, CA, USA) for 1 h and then incubated for 16 h at 4 °C with the following antibodies (all from Cell Signaling Technology, Danvers, MA, USA): GAPDH rabbit monoclonal antibody, clone 14C10, Cat. #2118; Akt rabbit monoclonal antibody, clone C67E7, Cat. #4691; Phospho-Akt rabbit monoclonal antibody (Ser473), clone D9E, Cat. #4060; p44/42 MAPK (Erk1/2) rabbit monoclonal antibody, clone 137F5, Cat. #4695; Phospho-p44/42 MAPK (Erk1/2) rabbit monoclonal antibody (Thr202/Tyr204), clone 197G2, Cat. #4377.

### 4.10. RNA Extraction and cDNA Generation

Solid samples were thawed, cut, and crushed using 1 mm and 2 mm zirconium oxide beads in the Bullet Blender (Next Advance, Troy, NY, USA). Liquid samples were thawed and washed by centrifugation 3–4 times in 4 mL Dulbecco’s Phosphate Buffered Saline (PBS; Biological Industries) until the fluids were pellucid. RNA from the cell lines and clinical specimens was then extracted using the Bio-Tri reagent (Bio-Lab ltd, Jerusalem, Israel) according to the manufacturer’s protocol. The RNA concentration was measured by a NanoDrop 2000 spectrophotometer (Thermo Fisher Scientific).

Prior to cDNA formation, 1 μg of the extracted RNA was subjected to DNA degradation by PerfeCTa DNase I (Quanta Biosciences, Gaithersburg, MD, USA). The cDNA was created by the qScript cDNA synthesis kit (Quanta Biosciences).

### 4.11. Quantitative Real-Time Reverse-Transcription Polymerase Chain Reaction (qRT-PCR) and RT-PCR

qRT-PRC was performed using the Fast SYBERTM Green Master Mix (Applied Biosystems by Thermo Fisher Scientific, Foster City, CA, USA) with specific primers in the CFX Connect Real-Time system (Bio-Rad Laboratories, Hercules, CA, USA). RT-PCR was carried out using Hy-Taq Ready Mix (2X) (Hylabs, Rehovot, Israel) with specific primers in PCR TOUCH T960 (Hangzhou Jingle Scientific Instrument Co., Ltd., Hangzhou, China). PCR products were loaded on 1.5% Agarose gel with 100 bp DNA ladder (GeneDireX Inc., Taoyuan, Taiwan). Gene expression was normalized to RPLP0. Primer sequences are detailed in Table 3.

### 4.12. Statistical Analysis

Statistical analysis was performed by applying the SPSS-PC package (v. 25). A probability of <0.05 was considered statistically significant. The Mann–Whitney U test or the Kruskal–Wallis H test was applied to the analysis of the association between LOC642852 levels and clinicopathologic parameters (for two-tier or three-tier analyses, respectively). For this analysis, clinicopathologic parameters were grouped as follows: age: ≤ 60 vs. > 60 years; effusion site: peritoneal vs. pleural; FIGO stage: III vs. IV; chemotherapy status: pre- vs. post-chemotherapy specimens; residual disease (RD) volume: 0 cm vs. ≤1 cm vs. >1 cm; response to chemotherapy: complete response vs. partial response/stable disease/progressive disease. Progression-free survival (PFS) and overall survival (OS) were calculated from the date of the last chemotherapy treatment/diagnosis to the date of recurrence/death or last follow-up, respectively. Univariate survival analyses of PFS and OS were executed using the Kaplan–Meier method and log-rank test. Multivariate survival analysis was executed using the Cox Regression Model. Platinum resistance was defined as PFS ≤ 6 months according to guidelines published by the Gynecologic Oncology Group (GOG), and progressive disease or recurrence was evaluated by the Response Evaluation Criteria In Solid Tumors (RECIST) criteria.

## Figures and Tables

**Table 1:** Samples analyzed in the generated clones

Protein	OVCAR-8	B9(1)	G9(3)	C10(4)	HeLa
ERK	1–3	4–6	7–9	10–12	13–14
AKT	15–17	18–20	21–23	24–26	27–28
GAPDH	29–31	32–34	35–37	38–40	41–42
p-ERK	43–45	46–48	49–51	52–54	55–56
p-AKT	57–59	60–62	63–65	66–68	69–70
GAPDH	71–73	74–76	77–79	80–82	83–84

**Table 2:** Clinicopathologic parameters of the HGSC effusion cohort (85 patients).

Parameter		Distribution
Age (mean)		38–81 years (62)
FIGO stage		
III		52
IV		33
Residual disease		
Primary debulking surgery (*n* = 56)	0 cm	8
	≤1 cm	20
	>1 cm	25
	NA	3
Interval debulking surgery (*n* = 25)	0 cm	10
	≤1 cm	6
	>1 cm	5
	NA	4
Only chemotherapy; no surgery		4
CA 125 at diagnosis (range; median)		11–43,800 (760) ^a^
Chemoresponse after primary treatment		
CR		40
PR		25
SD		5
PD		7
NA ^b^		8

Abbreviations: NA—not available; CR—complete response; PR—partial response; SD—stable disease; PD—progressive disease; ^a^ Available for 66 patients; ^b^ Not available (missing data or disease response after chemotherapy could not be evaluated because of normalized CA 125 after primary surgery or missing CA 125 information and no residual tumor).

**Table 3:** qRT-PCR primer sequences.

Name	Forward	Reverse
*LOC642852*	GCTTTGTTTCAGGGTTTTGG	AGGTGTGTCCCCAGCTTCT
*EGFR3*	CCACTCCCTCCATCTCCTG	GGATGCTGCCAAACTTGTTC
*TLE3*	AGTGTGGAAAAGGCAACCAG	GACACAGAGAGGGCAAGAGG
*RPLP0*	CCAACTACTTCCTTAAGATCATCCAACTA	ACATGCGGATCTGCTGCA

## Supplementary Materials

The following are available online at https://www.mdpi.com/1422-0067/21/15/5237/s1, Figure S1: KO in OVCAR-8 cells. Figure S2: Survival curves for OS (*n* = 655) and PFS (*n* = 614) in the analysis of the KM-Plotter database. Figure S3: FGFR3 mRNA levels in control cells and Kos.
