# Supplementary material for: The Biological and Clinical Role of the Long Non-Coding RNA LOC642852 in Ovarian Carcinoma
Source: Int J Mol Sci. 2020 Jul 23;21(15):5237. doi: 10.3390/ijms21155237 (PMC7432776; doi:10.3390/ijms21155237)
Supplement: Supplementary file 1 [file ijms-21-05237-s001.pdf]

International Journal of
Molecular Sciences

MDPI

Supplementary Materials

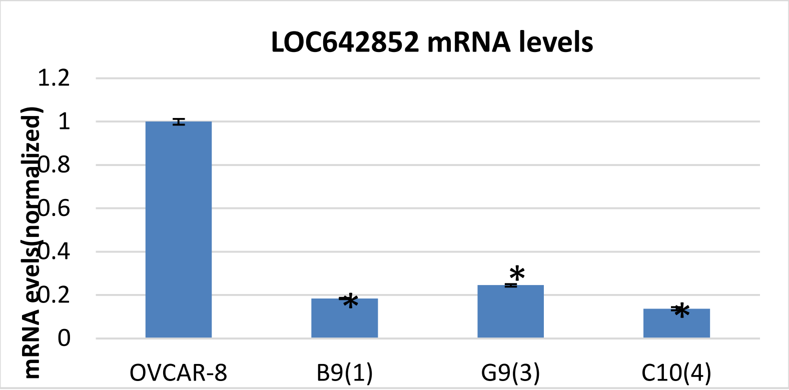

Figure S1: KO in OVCAR-8 cells. Expression was 18, 24 and 13% for B9(1), G9(3), and C10(4), respectively. * indicate $p < 0.001$.

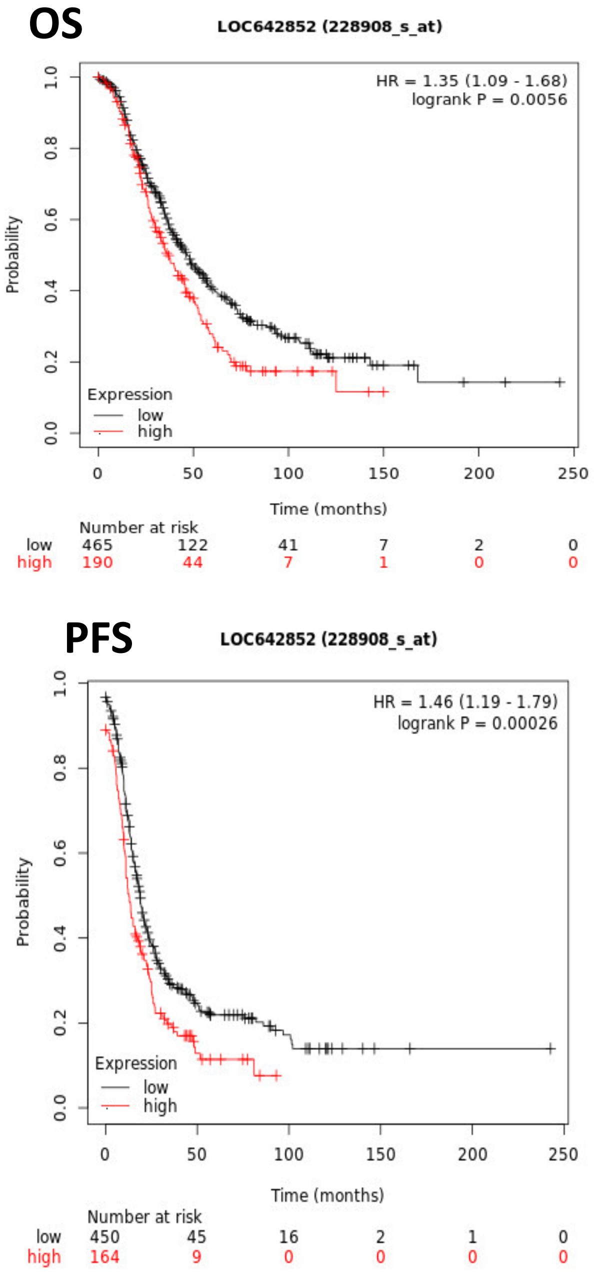

Figure S2: Survival curves for OS (n = 655) and PFS (n = 614) in the analysis of the KM-Plotter database.

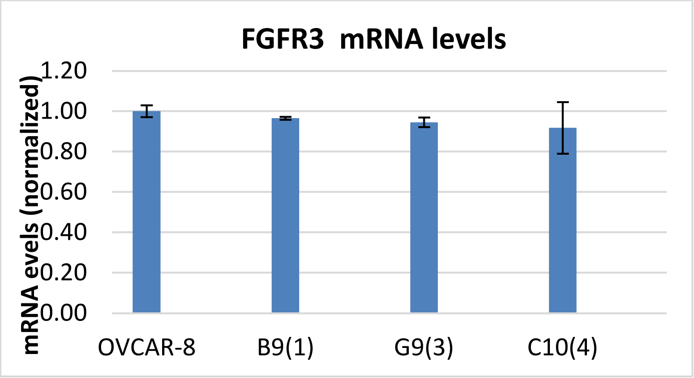

Figure S3: FGFR3 mRNA levels in control cells and KOs.
